# A Low Power IoT Sensor Node Architecture for Waste Management Within Smart Cities Context

**DOI:** 10.3390/s18041282

**Published:** 2018-04-21

**Authors:** Matteo Cerchecci, Francesco Luti, Alessandro Mecocci, Stefano Parrino, Giacomo Peruzzi, Alessandro Pozzebon

**Affiliations:** Department of Information Engineering and Mathematical Sciences, University of Siena, 53100 Siena, Italy; matteo.cerchecci@student.unisi.it (M.C.); francesco.luti@student.unisi.it (F.L.); alessandro.mecocci@unisi.it (A.M.); parrino2@unisi.it (S.P.); peruzzi26@student.unisi.it (G.P.)

**Keywords:** IoT, smart city, waste management, LoRa, LPWAN, energy saving, ultrasound sensor

## Abstract

This paper focuses on the realization of an Internet of Things (IoT) architecture to optimize waste management in the context of Smart Cities. In particular, a novel typology of sensor node based on the use of low cost and low power components is described. This node is provided with a single-chip microcontroller, a sensor able to measure the filling level of trash bins using ultrasounds and a data transmission module based on the LoRa LPWAN (Low Power Wide Area Network) technology. Together with the node, a minimal network architecture was designed, based on a LoRa gateway, with the purpose of testing the IoT node performances. Especially, the paper analyzes in detail the node architecture, focusing on the energy saving technologies and policies, with the purpose of extending the batteries lifetime by reducing power consumption, through hardware and software optimization. Tests on sensor and radio module effectiveness are also presented.

## 1. Introduction

Wireless sensor networks (WSN) are employed in the most diverse contexts, especially when the monitoring of random phenomena is requested. Indeed, by gathering data as much as possible, better forecasts could be performed: a WSN could be exploited with the purpose of achieving this task. In addition, they are also used whenever the monitoring of environmental parameters has to be accomplished. Nowadays, such parameters should be thought in a more broader sense: beside the standard atmospheric ones (such as temperature or moisture), also the level of smog or the level of traffic are purpose of study in urban environments. Beside those, also waste management has been for several years a hot topic whose optimization has been constantly increasing. This is the typical framework within which Smart Cities are defined: deploy sensor nodes forming a network, better if wireless, so as to monitor the occurrence and the extent of events in order to prevent car crashes, or to safeguard inhabitants health, or to manage assets and resources efficiently. Of course, these are only few instances since WSN suit well to disparate applications.

A WSN deployed in an urban scenario has to fulfill several requirements: sensor node casing has to be designed in a suitable manner so to protect the node from atmospheric agents, real time monitoring should be ensured if needed, power consumption must be optimized in case the node has to be installed in places not covered by the electrical grid or by renewable energy sources. The latter reflects the case of study of this paper since the node has to rely only on a limited source of power provided by four AA lithium batteries, as it will be seen later on.

This paper is composed as follows: in Section 2 we propose the state of the art of the employment of WSN within Smart Cities and of the solutions to the problem of solid waste management too while in Section 3 we present some related works regarding energy-saving policies for sensor nodes. In Section 4 the proposed node architecture is explained while in Section 5 the energy-saving policy chosen for the sensor node as well as an estimate of the consumption are shown. In Section 6 the tests performed are presented, while in Section 7 some concluding remarks and future works are highlighted.

## 2. Smart Cities and Waste Management

Many papers describe the strict correlation between Smart Cities and WSN focusing on how this technological framework can be exploited to develop interconnected intelligent systems. Moreover, if the data gathered by the network are made available on the Internet, then the IoT framework (which, nowadays, is a hot-topic) is encountered.

For instance, in [1] such relation is highlighted. The authors report a list of applications encompassed by the concept of Smart City: structural health of buildings, air quality, noise monitoring, traffic congestion, city energy consumption, smart parking, automation and salubrity of public buildings, smart lighting and finally waste management. Then, they present how these services are performed within the whole picture of the Padova Smart City project whose aim is to develop an IoT island in the city of Padua, Italy.

Within the context of air quality and pollution monitoring [2] gives an interesting overview of the problem and how to solve it by means of a wireless sensor node that is part of an IoT system. The authors propose an interesting energy-saving policy which controls the duty-cycle of the whole sensor node. This technique and the one shown in Section 5.1 are developed in a pretty similar fashion. However, it relies on a rechargeable battery via a photovoltaic cell and, as it will be described in Section 4, renewable sources of energy cannot be exploited in the proposed scenario. Reference [3] proposes an IoT infrastructure for Smart City and examines the case of study of the noise monitoring, while [4] shows a solution for long term structural health monitoring of buildings reporting the case of study of a church sited in L’Aquila, Italy, which is a city recently struck by an earthquake.

There exist several useful services that can be implemented in Smart Cities by using WSN-based monitoring systems. They span throughout many application fields, such as environment, health, industry and home automation. Moreover, in recent years, many enabling technologies (like GPRS, RFID, GPS, WiFi) have been investigated in order to solve issues related to the various phases of solid waste generation, monitoring and management: these matters are often critical in terms of both operating costs and environmental quality.

This paper deals with a WSN solution to face the solid waste monitoring by using an innovative IoT node architecture so as to monitor the filling of garbage bins. In particular, this case scenario is only a possible test-bed since the optimized implemented solution allows a node to reduce power consumption, therefore it may be applied to many other working fields.

In the literature, several papers [5,6,7,8,9,10,11] propose solutions using WSN for waste management. Some researchers proposed a WSN and a web browser [5] for monitoring the bin status: this is a crucial parameter in this application field since if a bin is partially filled then it does not need any depletion. As a consequence, human intervention would be limited causing a reduction of the cost involved by solid waste collection. The authors opted for multiple communication technologies within the WSN. One of these is the GSM/GPRS, therefore such a paradigm requires high running costs. Moreover, they did not account for any energy-saving policy claiming that it should be a future works target.

Solution [6] was developed by implementing Vehicular ad hoc networks (VANETs), providing a model in which the garbage collection is monitored in real time: each trash bin is equipped with a sensor node that continuously senses its status and when it is full it sends a signal to the nearest garbage collecting vehicle (hence, each of them is a WSN node as well). Then the vehicle will collect the garbage and will move back. In so doing, garbage collection from bins is made dynamically and it results a much more efficient solution in comparison with the traditional bins emptying based on planned collections. A drawback of this solution is that two LASER diodes, which in turn act as transmitter and receiver, are exploited for sensing the filling level of trash bins. As it will be explained in Section 4.2 it could be an unreliable method. Beside this, the authors did not adopt any energy-saving policy at all. Moreover, in [7] efficient routing algorithms are advised as well as real-time waste related information. In particular, the filling level of bins can be sent to a monitoring and Decision Support System (DSS) owing the storage of historical data that can be exploited to predict waste bins filling rates.

In the IoT applications, many designers and developers opted for microcontroller boards (for example Arduino boards) or Systems on Chip (SoC, such as Raspberry Pi, BeagleBone, Lopy) as platforms to provide sensor nodes with Internet connection: this choice is often very complex, due to the fact that a trade-off between costs, performances and functionalities is needed for each particular application project.

Many enabling technologies are used to setup smart platforms, which are capable of collecting and managing data, that deal with problems related to waste management. Some examples are: environment pollution due to overflowed trash bins that causes human diseases, increasing number of insects and mosquitoes. Emerging technologies and innovative devices are used to optimize these solutions, even though ZigBee and GSM technologies are still a valid choice, especially if combined with microcontrollers or SoCs. Some IoT smart waste management systems combine the strengths of WSN with the high performances of SoCs [8]: the embedded board acts as a node in a WSN based on the ZigBee protocol and consisting of a coordinator and sensor nodes distributed in the test area. The coordinator collects data from sensor nodes and then sends it to the communication board, that stores collected data in a database, analyzes it and provides a user interface to monitor them. Each of the trash bins is equipped with an Arduino UNO board. This directly implies an increase of both costs and consumption. In addition, the authors choose a network architecture that relies on a coordinator node for which a Raspberry Pi board is exploited involving higher costs and consumption as well.

Smart garbage bins [9] allow authorized people to acquire information concerning the filling level, through ultrasonic sensors placed on the bins and by using a GSM-equipped microcontroller that sends data to a control station. A similar study [10] shows how it is possible to acquire useful data (garbage level, wet garbage and toxic gases detection) from a set of sensors (ultrasonic, moisture and gas sensors) installed within the bins and connected to a microcontroller which sends data covering a long distance through a ZigBee transmitter, also sending SMS to mobile devices by using a GSM module. Once again, these two last solutions do not suit well to the context of this paper since GSM entails pretty high running costs. Reference [11] shows a sensor node for monitoring the filling level of waste bins equipped with RFID technology, that could be a viable solution due to its robustness and cheapness. Unfortunately though, it cannot provide a communication range as large as the one ensured by Low Power Wide Area Network (LPWAN) technologies (as described in Section 4.1).

A recent study [12] proposes how to implement a smart garbage management IoT system combining sensors and Raspberry Pi, while a well-performed survey [13] about smart garbage management in cities using IoT suggests many other ideas that may be implemented.

## 3. Related Works

One of the targets of this study is to design a node whose power requirement is limited. If such a characteristic is satisfied, then a direct consequence would be an extension of the batteries lifetime which directly entails a minimization of human intervention to replace them. However, the fulfillment of this project specification is pretty challenging during the design phase of a wireless sensor node since it is mandatory to include a data transmission module. This component is one of the most power-hungry though, therefore at least one energy-saving approach (i.e., a strategy), or even more than one at the same time, must be adopted.

Concerning the energy-saving aspect, in references [14,15] event driven architectures are exploited in the Smart Cities perspective. Such an option has been avoided since the most suitable event for the application scenario (i.e., a waste disposal) is not reliable. Indeed, the realization of this event could have a relatively high frequency implying an almost constant powering of the IoT sensor node. That is the reason why a time-based policy is preferable.

From the point of view of the consumption optimization, the core process resides in the employment of both a low power transceiver and a low power microcontroller since they are the components that most affect the available energy budget. With respect to consumption optimization, some papers, such as [16,17], treat the issue proposing some studies and ideas to improve the node lifetime.

In general, the most immediate energy-saving strategy obviously is to provide the sensor node with an energy harvesting solution [18]: the most common is the use of photovoltaic panels [19] but also other strategies have been proposed [20,21]. An hydroelectrically powered sensor node was already discussed by the authors in [22]. While all these solutions are able to continuously power the monitoring system, they are unsustainable in term of costs. Even the cheapest energy harvesting solution has costs in the order of tens of euros, in the same order of magnitude of the overall cost of the components of the proposed node. Moreover, as it will be discussed in Section 4, the application scenario is not suitable for the use of renewable energy sources.

A more cost-effective way to improve energy efficiency is to control the duty cycle of the node in an adaptive fashion, as it is shown in [23] where the authors present a control law for the duty cycle of the sensor node supposing that it is able to harvest energy form a certain source within the application scenario and that it relies on a rechargeable battery. The aim of the control law is to maintain a constant charge level of the battery by adjusting in real-time the duty cycle. Unfortunately though, the sensor node we are presenting cannot make use of any harvestable source of energy as it will be seen later on. Other methods based on mixed techniques of energy management are described in [24,25,26]. For instance, reference [24] gives a dichotomous division of energy management of sensor nodes: duty cycling and adaptive sensing. Concerning the latter, a classification of the strategies based on the way sampling is performed is provided pinpointing three macro-strategy paragons: hierarchical sensing, adaptive sampling and model-based active sensing. The last named could be one of the targets of future work related to the one we are showing. Indeed, such a technique develops a predictive model of the sensed phenomenon starting form set of data preliminary acquired. Those data could be the ones gathered with our prototype. Once the model is accomplished, energy consumption could be limited by sampling whenever it is supposed to be necessary according to the prediction of the model rather than on a periodic basis as it is done by means of duty cycling. Moreover, new acquired data could be useful to tune the predictive model as well.

Some inspirations from which one can take advantage could be found in [25]. A heterogeneous multiprocessor sensor node is one of those, whose general idea is also exploited in our paper. Indeed, a sensor node generally has a workload that can be split into two rough parts depending on the amount of fulfilled operations. That amount directly implies a proportional power consumption resulting in a low workload period and a high workload one. Therefore, a sensor node could be equipped with two microcontrollers/microprocessors (both of them share elements like sensors and actuators, communication modules and power supply) each of which is in charge of managing the appropriate workload period (i.e., during the lower one the energy-thriftier microcontroller runs keeping turned off the energy-hungrier one and viceversa). As it will be verified in the rest of this paper, we commit to that paradigm: the two workload phases are managed by a microcontroller (for the higher one) and by a counter (for the lower one). Even though a counter is not an actual microcontroller, it has been chosen since no computational power is needed during the relative workload period.

The survey [26] exposes different strategies to reduce the consumption of sensor nodes that rely on a limited budget of energy. Among many alternatives, the authors also point out the duty cycling method claiming that it has a main impact on energy waste sources. In particular, such a method is at the same time more effective on multiple causes of energy waste (like the sensing and processing phase, the communication phase or the idle phase) than other methods (like the topology control of the network or energy efficient routing algorithms). Of course, by combining those methods better results would be achieved. However, the sensor node we are proposing has not been included within a multi-hop network (it will be explained later on that our node was successfully tested by setting up a point-to-point wireless communication with a remote gateway, nonetheless it is expected to be part of a single star wireless network in the future), therefore only the management of duty cycle has been accounted.

Other energy-saving techniques reside in thrifty communication protocols from the consumption point of view: at the network layer, by using energy aware routing algorithms [27,28,29,30,31,32,33,34,35,36,37], and at the medium access control layer as well [38,39,40,41,42,43,44,45]. Also the network topology, as well as the node position within it, influences energy consumption. Regarding the former, a sparse topology could be an energy efficient solution [46]. Briefly, the idea proposed in this paper is to turn on each sensor node only when it detects an occurrence of the monitored event and then to turn it off once the transmission phase is completed; of course, some circuitry capable of detecting such an event should be constantly turned on and therefore it has to be carefully designed so as to limit its energy requirement. Regarding the position of the node within the network, it is well known that the higher the amount of data processed and transmitted by the node, the more power it requires. For instance, in a star network topology the central node requires much more power than the other ones. On the contrary, a node of a chain-type network with multi-hop communication methods requires more power as it is closer to the base station, since it is subject to a more intense data traffic compared with the one of the most peripheral nodes. This issue is addressed in several papers like [47,48,49,50,51]. There are also some surveys and papers that investigated power optimization algorithms for WSN [52,53,54,55,56]. Basically, they pointed out multi-objective paradigms, and some open problems as well, in order to improve lifetime in WSN. Due to all these reasons, a single star topology, rather than a chain-type one, will be preferred for the future realization of the WSN discussed in this paper, having replicas of our sensor node as components. Indeed, the central node will be conceived bearing in mind the availability of the electric grid. This condition will be easily met due to the logistic of the application scenario. Adopting such a network topology, most of the solutions described in these papers are of few interest for the proposed architecture.

With the purpose of reducing energy consumption, it is fundamental to revise the single components of the node architecture. Usually, the main building blocks are: a microcontroller (or a microprocessor), a communication module and some sensors. The choice of each part should be done in such a way to limit its individual energy requirement. For instance, it is far better to use passive sensors instead of the active ones, or it is better to use a low power transmission module capable of covering only the transmission distance needed. An interesting review of this topic is reported in [57], while in [58] some techniques to decrease power needs and increase energy efficiency of a wireless sensor node within the Internet of Things (IoT) context are proposed. Both of those studies provided interesting suggestions and advice during the design phase of our sensor node, especially for what concerns the alternatives from which we chose its building blocks.

In the design phase of a WSN sensor node that has to be employed in all these contexts, one of the most important concepts to take into account is power management. A possible approach is to optimize each component of the node: the elaboration device (microcontroller, SoC), sensors and their conditioning circuits as well as the transceiver module for wireless communication. Due to the fact that sensor nodes are often powered through constrained power sources, like batteries, their consumption needs to be reduced. For this reason, the components of the node architecture have to be investigated so as to identify all their energy requirements needed for achieving both the sensing operations (with the related data processing) and the radio transmission. Indeed, a viable solution consists of equipping nodes with low power technologies: BLE (Bluetooth Low Energy), 6LoWPAN (IPv6 over Low power Wireless Personal Area Networks) or the emerging LPWAN solutions like the LoRa and SigFox protocols. In the following section, an optimized sensor node architecture based on one of these last technologies, i.e., the LoRa protocol, will be proposed.

Eventually, this section is concluded by highlighting some common aspects as well as some improvements in comparison with some related works, which span among different application scenarios, belonging to the literature. Let us start by citing [59] which shows how the sand transportation due to wind on a coastal scenario could be measured via a WSN having heterogeneous sensor nodes. In particular, one of those nodes is a wireless battery-powered scale placed on the bottom of a sediment trap whose estimated lifetime is of 14 days. Its main components are: a 3000 mAh battery pack, a linear voltage regulator, a load cell and its conditioning circuit and a ZigBee transmission module. Since sediment measurements are periodically performed and transmitted, the sleep mode for the transmission module is enabled whenever data are not required. Moreover, since data processing is remotely accomplished, then a microcontroller is not necessary. In spite of the fact that our sensor node is equipped with a microcontroller, it has a noticeably higher lifetime (as it will be seen in Section 5.2) mainly due to these reasons that involve a consumption reduction: a step-down voltage regulator is employed in place of a linear one (see Section 4.4), an energy-saving policy capable of turning off the energy-hungrier parts of the system is adopted rather than exploiting a simple sleep mode (see Section 5.1) and a higher amount of energy is available thanks to the usage of bigger capacity batteries (see Section 5.2). In [60] a sensor node for the measurement of the water level within the context of irrigation networks is presented. Such a node is mainly made up of the following components: an ultrasonic sensor and a temperature sensor (and their front-end electronics), a microcontroller, a GSM/GPRS module and a 14,000 mAh battery. The authors estimated the sensor node lifetime as 691 days by actuating a similar policy compared with the one that will be described in Section 5.1: all the elements are turned on only when necessary apart from the microcontroller which operates in a sleep mode when it is idle. That figure is higher than the one that will be found out in Section 5.2 due to the usage of a battery having a far bigger capacity than the one employed in the solution we are proposing. Unfortunately, suchlike battery was not available during the realization of the sensor node we are showing, otherwise its lifetime could have roughly been four time longer. The study [61] reports a wearable sensor node for indoor environmental monitoring whose aim is to supervise the level of harmful gases within industrial scenarios. That node is formed by the following main building blocks: a microcontroller, a temperature and humidity sensor and a CO_2_ concentration sensor (along with their conditioning electronics), a ZigBee module, a couple of voltage regulator and a 800 mAh battery. The wearable node lifetime estimate is slightly more than 5 days. This result is pretty scant with respect to the one of the node we are describing (see Section 5.2) principally owing to two reasons: since the battery has to be a small-sized one in favour of wearability, only a small amount of power is available; the wearable sensor node is equipped with a not so optimal energy-saving policy, indeed none of the components are switched off every time that they are not necessary (only the microcontroller operates in sleep mode when it is idle whose waking signal is provided, as an interrupt, by the ZigBee module, therefore the latter has to be constantly turned on).

## 4. Node Architecture

This paper is focused on a solution for the real-time monitoring of the filling level of trash bins, placing the optimization of power consumption at the bulk of all the design activity. Beside this primary target, cost containment has been considered as the secondary one. The requirement of low power consumption implicitly implies that no connection to the electrical grid will be available at the deployment site of the node. Therefore, the node is expected to be powered through either energy harvesting solutions (e.g., solar cells) or batteries. The use of renewable energy sources is expected to double the overall cost of a basic IoT node like the one proposed in this paper, and the deployment of these solutions may not always be possible in highly urbanized areas (e.g., trash bins are often employed in historical centers where in narrow alleys direct sunlight is available only few hours per day or even never): for this reason, the architecture proposed in this paper has been customized on battery-powered IoT nodes. In particular, power is provided by four 1.5 V AA lithium batteries whose capacity is 3500 mAh.

Since the crucial component affecting power dissipation in sensor nodes is the transmission module, such a design activity will be focused not only on the structure of the sensor node but also on the definition of the network architecture. To this end, the identification of an efficient data transmission technology is the square one towards the realization of an energy efficient sensor node. Beyond this, an investigation in order to determine which is the necessary amount of computational power should be carried out. For instance, if data processing is required on board of the sensor node, then a powerful computing unit is needed; this entails bigger consumption though. On the other hand, if data acquisition is the only operation that the node has to perform, then such a constraint could be relaxed by employing simpler devices. Hence, the choice of the microcontroller/microprocessor ensuring the best trade-off between energy efficiency and complexity has to be selected. Eventually, sensors have to be chosen looking for the best compromise between the accuracy of the measured data and the required energy to perform such task, thus privileging the use of passive sensors since power hungry sensors may become the crucial factor for energy consumption.

Once identified the main components of the node, the design has to be focus on their integration, choosing the best electronic components for their interconnection and implementing energy saving policies through the use of procedures and technical solutions that will allow to turn off the node, or parts of it, when they are not used. Such energy-saving policy will be introduced later on in Section 5.1.

The proposed IoT node was designed following this kind of approach, starting from the choice of the basic components: a single-chip microcontroller (ATmega328/P by Atmel (San Jose, CA, USA)) rather than a general purpose single-board microcontroller was employed, while for data transmission the choice fell on the LoRa technology, also taking into account an overall network architecture shaped on a Smart City scenario. Finally, a low cost and low power sensor was chosen, providing the best ratio between measurement accuracy and energy efficiency.

**Figure 1:**
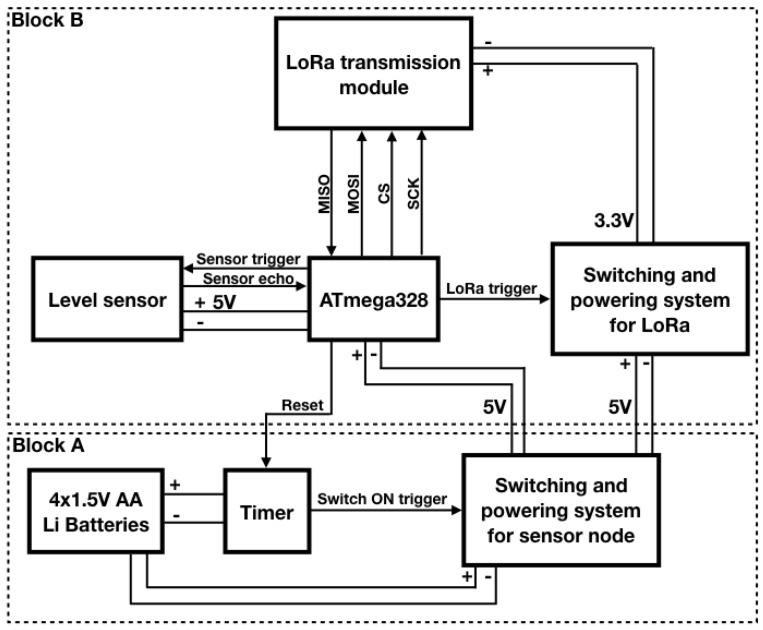
Block diagram of the proposed IoT node.

**Figure 2:**
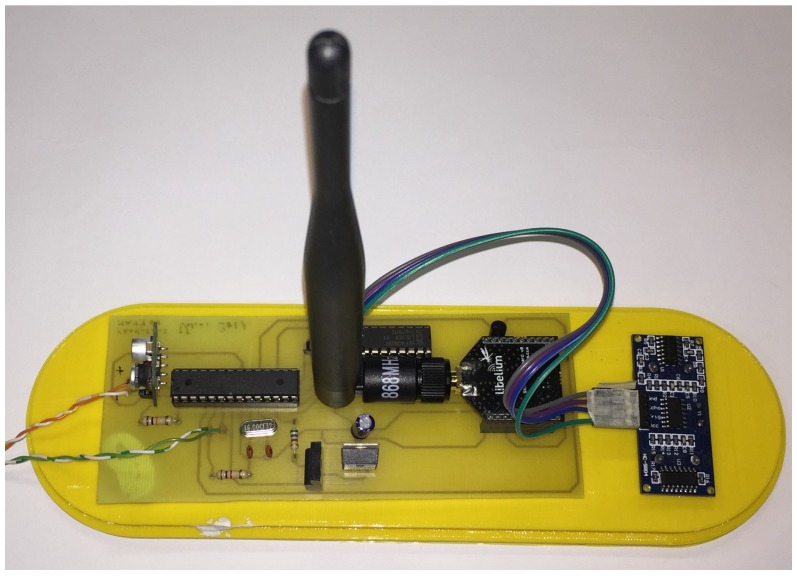
Circuit of the IoT sensor node.

Once the single components were selected, then the design was focused on their integration into a single board, embedding additional components required for energy management. The final node architecture is described by the block diagram shown in Figure 1: in addition to the main components, two switching circuits turning off respectively the LoRa radio module and the node in its entirety were added in order to implement duty-cycling policies. In particular, a timer controls the latter switching block. The diagram is divided into two main blocks: Block A is in charge of managing the duty cycle of the IoT node, and Block B measures the filling level of the bin and then transmits such a data. The structure of each single block as well as its functioning will be described in detail in the following subsections. Ultimately, in Figure 2 the final realization of the IoT sensor node is shown: the topmost outgoing twisted pair wires are exploited to connect the AA lithium batteries while the other pair was temporarily exploited only for the very first tests.

### 4.1. LoRa Transmission Module

As anticipated in the previous section, the data transmission module plays a significant role in power consumption optimization, therefore it has to be carefully chosen. Since replicas of the proposed IoT node are expected to be deployed outdoor, in large quantities and across large areas, only wireless solutions will be taken into account: indeed, it is obviously unthinkable to have a wired connection (e.g., Ethernet) at each trash bin. First of all, wireless connections can be subdivided according to their data transmission ranges. In particular, for IoT communication infrastructures, they can be summarized in three categories:

- **Local Area Networks:** based on different technologies and protocols, these networks have limited transmission ranges, usually lower than 100 m: this means that the data collection centre has to be located in the same area of the nodes. Alternatively, a sort of gateway node is required, which is in charge of receiving data from the nodes and forwarding them to a remote data collection centre through the Internet. The most common technologies belonging to this category are Bluetooth Low Energy (BLE), ZigBee and WiFi.
- **Wide Area Networks:** this category encompasses the emerging LPWAN Sub-GHz technologies like LoRa and SigFox, that provide transmission ranges that can reach 20 km in line-of-sight. In urban areas these technologies are able to transmit data even at a 3 km distance, representing the ideal solution for Smart City scenarios. Indeed, no middle layer is required between the nodes and the data collection centre: a single gateway can be able to collect data from large quantities of nodes distributed in wide areas: tests on transmission ranges proved that a single gateway could be able to cover a medium-sized city centre [62]. However, for larger areas more gateway nodes could be set up with a limited impact on costs and complexity of the network.
- **Global Area Networks:** they allow the transmission of data anytime and anywhere on the Internet as long as there is network coverage. This category encompasses all the cellular networks as well as more complex technologies like satellite communications. 2G (GSM, GPRS) and 3G (UMTS) radio modules are widely used for IoT nodes as well as 4G (LTE)-based solutions. Among the latter, the most promising one is the Narrow Band IoT (NB-IoT). All the modules available on the market are currently quite expensive and relatively power-hungry. However, with the emergence of the upcoming 5G technologies, new scenarios are expected to open for IoT architectures, favored by the promise of low costs and reduced energy consumption. Nonetheless, being SIM-based systems, all these technologies are characterized by fixed service costs that may be significant in case of IoT infrastructures constituted by large quantities of nodes.

Since all these technologies represent good alternatives for the realization of IoT nodes, the choice for the best one has to be made according to the specifications of the deployment scenario that has to be carefully studied. Regarding the one proposed in this paper, i.e., a Smart City solution for the real time monitoring of filling level of trash bins, the following assumptions can be made:

- The nodes are expected to be deployed in large quantities since even in a medium-sized city the number of trash bins amounts to some hundreds. Therefore, their cost has to be kept as low as possible and, at the same time, the maintenance operations have to be minimized;
- The area to be covered is large, some tens of km^2^;
- The sampling rate can be kept very low. One sample per hour may be sufficient for the purposes of the whole monitoring infrastructure.

Following these simple requirements, it is possible to determine the advantages and drawbacks of the three network categories listed before for the proposed scenario:

- **Local Area Networks:** since the area to be covered is notably larger than the transmission range of these technologies, ad-hoc architectures should be studied. Using WiFi or BLE would require the set up of a large number of gateway nodes or the presence of a capillary WiFi infrastructure. This would notably affect the overall cost of the deployed infrastructure. Another option could be the deployment of a mesh ZigBee (or BLE mesh) network: mesh networks are not energy efficient since they allow a considerable reduction in power consumption of End Device nodes but not for Router nodes that have to be always switched on. This means that, even though their overall current absorption is on average quite low (∼40 mA [63]), their lifetime when powered by batteries is however in the order of some days.
- **Wide Area Networks:** LPWAN solutions are intrinsically characterized by low power consumption [64]: their larger transmission range allows to set up star or multi-star networks with few access points, permitting the sensor node to exploit duty-cycling policies that allow to turn off the node for prolonged periods. In the case of medium-sized cities a single gateway node could be sufficient for the whole network. Finally, the cost of LPWAN radio modules is extremely low consenting their use in large quantities without the additional costs as it would be in the case of SIM-based services.
- **Global Area Networks:** this solution is the easiest one to be deployed since it does not require access points or the realization of an ad-hoc infrastructure. Unfortunately, it has many other critical aspects, the first of which is the cost: GPRS/UMTS modules, even the cheapest ones among these technologies, are still more expensive than other solutions. Moreover, they are SIM-based thus they require the payment of a subscription that, when planning to deploy thousands of nodes, becomes not negligible. In addiction to it, also the question of power consumption has to be taken into account: the average current absorption of UMTS is ten times greater than the one of ZigBee or LPWAN modules [65], making it not suitable for low power applications.

All the previous considerations suggest that the best choice for the proposed scenario is represented by Wide Area Networking solutions, since they provide the best ratio in terms of transmission range (and then complexity of the network to be deployed), costs and power consumption (and then lifetime). Thence, the choice for the transmission module to employ fell on LoRa technology and LoRaWAN protocol: this solution allows the development of a fully customized network (unlike the SigFox technology which is based on a proprietary cloud platform), that is widely employed and then well-known in terms of performances and reliability. Moreover, a wide range of devices are already available on the market having low prices, under few euros, hovering around 15÷25 euros.

The radio module used for the development of the node prototype is a Libelium SX1272 LoRa module [66]: this device was chosen due to its ease in the interfacing with an Arduino UNO board (used for the first tests) through an ad-hoc Multiprotocol Radio Shield. Moreover, following a set of tests among similar platforms (e.g., [67]) performed in the historic centre of Siena, Italy, it proved to provide the best results in terms of transmission ranges, reaching 3 km in urban areas. Table 1 shows the main features of the radio module.

Since the LoRa module is controlled by the microcontroller, then the Serial Peripheral Interface (SPI) bus is exploited in order to manage the communication between these two components. Therefore, these devices communicate synchronously in full-duplex in a master-slave fashion where the microcontroller acts as the master. As it can be seen in Figure 1, four signals are reported:

- MISO (Master Inpunt Slave Output) is used by the slave to transfer data towards the master;
- MOSI (Master Output Slave Input) is used by the master to transfer data towards the slave;
- CS (Chip Select) is used by the master to select the slave to communicate with;
- SCK (Serial Clock) is used by the master to synchronize the communication.

Such signals are exploited to fulfill the data transmission of the sensed trash level: the microcontroller selects the LoRa module via CS and it starts the clock signal via SCK; the data to be transmitted is sent through the MOSI so that the module is able to transmit it and when the transmission is over the module notices it to the microcontroller via the MISO.

### 4.2. Filling Level Sensor

Since the main purpose of the IoT node is to measure the filling of trash bins, the most suitable sensor to perform this measurement has to be chosen. In order to define the features of the sensor, the following assumptions can be made:

1. The filling level of trash bins cannot be calculated measuring the weight of the introduced waste. Indeed, a heavy weight does not always mean a large volume. Metallic materials can be very heavy yet leaving the trash bin almost empty, while a large amount of paper can totally fill the trash bin though being very light;
2. The inner part of a trash bin is subject to a high level of fouling, then preventing the use of optical sensors;
3. Trash bins are used for both very small and large objects. This means that solutions based on the count of thrown items do not provide an accurate filling estimate.

In addition to all these requirements, two further features have to be provided by the sensor. The first one concerns the cost. Indeed, the sensor is expected to be cheap and since the node has to be deployed in very large quantities (even some thousands), then its overall price is expected to be in the order of few tens of euros. This means that the cost of the sensor should be at least lower than 5 euros. The second requirement concerns the energy consumption. The current absorption of the sensor is expected to be the lowest possible. As it will be explained later on (see Table 2), all the employed components account for a maximum 28 mA absorption. This should be considered as an upper bound also for the sensor, even if a lower value is expected to improve the overall node efficiency.

All these constrains significantly narrow the range of possible solutions: all weight sensors, regardless of the type (piezoelectric, load cells, etc.), can be excluded due to the first constrain, while the second one leads to the exclusion of optical sensors like cameras (that may be also excluded due to the higher costs and power consumption) and infrared sensors that could have been used to measure the proximity of the trash level to the top of the bin. The last constrain also leads to the exclusion of magnetic or mechanical sensors that could have been applied to the opening of the bin counting the number of openings.

Following all the previous considerations, the choice for the best solution fell on ultrasound proximity sensors. These devices measure the distance from a target through an ultrasound emitter (the trigger) and a receiver (the echo): the trigger emits a discontinuous ultrasound signal that hits the target and then it is reflected backwards. The echo detects the reflected signal: by the knowledge of the time interval between the emitted and the reflected signal, and through the value of the speed of sound in the air, it is possible to calculate the distance from the sensor to the target which can be easily translated into the filling level of the bin.

These sensors are compliant with all the previous requirements:

- They do not measure the weight of the trash but, if placed in the internal upper surface of the bin, they measure the actual level of the garbage layer by calculating its distance from the top of the bin;
- They are immune to fouling since ultrasounds are not perturbed by the presence of dirt or dust on the sensor surface;
- Since they detect the actual volume occupied by trash, they measure the filling level regardless of the number of introduced items;
- They have very limited costs (under 2–3 euros) and a limited current absorption (usually <15 mA; there exist also sensors absorbing 3–4 mA but unfortunately they are significantly more expensive, though).

Due to the wide range of sensors available on the market, the one used for the prototype was chosen putting the cost requirement in first place. For this reason an HC-SR04 sensor was employed: this device is available on the market at even just 1 euro and, according to its datasheet [68], it is characterized by a 15 mA working current. The HC-SR04 sensor is provided with 4 connectors: the $V_{CC}$ and Ground pins that allow to power it and the Trigger and Echo pins that are used to generate and sense the emitted and reflected ultrasound signals respectively. Once the time interval *t* between signal emission and receiving is measured, the distance *d* can be calculated according to the following formula:

(1) $$d=\frac{v_{spread}\cdot t}{2}$$

where $v_{spread}$ is the ultrasonic spread velocity in the air. In fact, such a speed depends on temperature: at a temperature of 15 °C, for instance, the speed is $v_{spread}$ = 340.7 m/s. Formally, considering that *T* is the temperature at which the sensor is exposed (expressed in °C), then the actual spread velocity can be evaluated as follows:

(2) $$v_{spread}=331.4\;\frac{m}{s}+0.62T.$$

In other words, according to Equation (2), if a variation of 20 °C occurs (which, incidentally, is feasible) then it would cause a variation of speed less than 4%. Since a bin is approximately 1 m tall, then such a variation could be negligible when the filling level is measured. Obviously, the filling level raises as the bin is filled up (it could be thought as the bin would get shorter), hence such a variation constantly becomes more negligible, if finer estimates are needed, then a temperature sensor could be embedded within the node so as to compensate the changing of the spread velocity. Anyhow, for the prototype described in this paper the temperature compensation is avoided due to the reason just explained. Eventually, Equation (1) is implemented by the microcontroller of the IoT node which, as it will be covered in detail soon, is also in charge of supplying the sensor only when it is strictly necessary.

### 4.3. ATmega328 Microcontroller

The ultrasound distance sensor, as well as the LoRa module, requires the presence of a microcontroller unit to operate: this component too has to be chosen on the base of the best compromise between cost, power consumption and efficiency. Since most part of low power microcontrollers have current absorption around few mA, the impact of this component on the overall power consumption of the node can be considered negligible, allowing a greater degree of freedom in the choice.

As a consequence, the first prototype of the node was set up by using an Arduino UNO development board. This platform allowed a plug-and-play solution for the integration of the LoRa transmission module: indeed, through an Arduino Mutliprotocol Radio Shield it is possible to integrate the LoRa module directly on board, possibly combining it with other wireless communication modules like ZigBee or Bluetooth. The Arduino UNO board was also chosen for the presence of 6 analog and 13 digital pins, allowing the direct connection of a wide range of sensors, and of a 3.3 V pin, a 5 V pin and 3 Ground pins. All these ports enable the direct connection of the HC-SR04 sensor since, as described in Section 4.2, it requires 5 V and Ground for powering and two digital pins for the signal.

**Figure 3:**
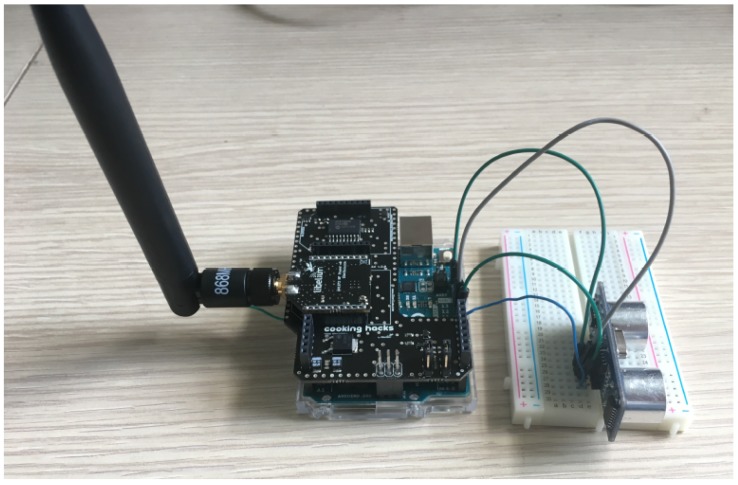
First prototype of the node.

The first prototype (Figure 3) was then realized by simply integrating the components listed before: it allowed to test the operation of the whole node. Following these first tests, the operation of the HC-SR04 sensor as well as its effectiveness in measuring the filling level of the trash bin, and of the LoRa transmission module, was proved. Additional details on the test phase will be presented in Section 6.

Since the proposed sensor node has only to read an analog value from a sensor, calculate the filling level and transmit it through a radio module, the use of a general purpose single-board microcontroller (like the Arduino UNO) can be considered redundant. Indeed, this kind of board is provided with a wide range of analog and digital inputs as well as LEDs and other components that are useless for the proposed scenario. Moreover, single-board microcntrollers have higher current absorptions if compared with the corresponding microchip microcontroller. For example, while current absorption of the Arduino UNO board is around 40 mA, the absorption of the ATmega328 microchip (the microcontroller on board of the Arduino) is only 10 mA. Since the average current absorption of low power microcontrollers when active is generally low, in the order of few mA, the choice of the microcontroller to be employed was based more on its technical features and ease of use rather than on its actual power consumption. For this reason the choice fell on the ATmega328/P microcontroller, whose datasheet is reported in [69], since it is the one on which the Arduino UNO board is designed and did not require any additional programming and hardware compared to the prototype made with Arduino. This single-chip microcontroller requires a few additional circuitry to run, like resistors and capacitors, and a quartz crystal so as to set its clock frequency. Moreover, this microcontroller can be purchased with the Arduino bootloader already set on it, making it easily programmable with the same firmware developed for the Arduino board. As it will be described within Section 5.1, the microcontroller firmware plays a fundamental role in implementing the energy-saving policy: it is in charge of switching on the level sensor as well as the LoRa module only for the required time respectively for the measure and the transmission, involving a reduction of power absorption. Finally, by means of a firmware instruction, the microcontroller shuts down the whole Block B of Figure 1.

### 4.4. Block A

Power supply is provided by four 1.5 V AA lithium batteries, hence the node is provided with an amount of 6 V, and considering that the microcontroller and the transmission module require lower supply voltages, 5 V and 3.3 V respectively, it is mandatory to include voltage regulators within the node. For this reason, two voltage regulators are employed: a 5 V regulator which powers Block B of Figure 1 and a 3.3 V one which powers the LoRa module. The latter is within the Switching and powering system for LoRa block and therefore it will be introduced in the relative Section 4.5.

**Figure 4:**
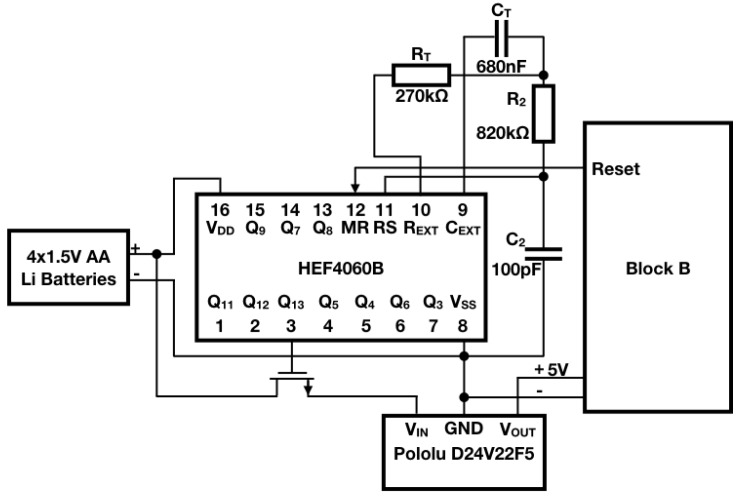
Block diagram of Block A part of the IoT sensor node.

Block A is characterized by the block diagram presented in Figure 4. The duty cycle of the sensor node is managed by an integrated circuit: the HEF4060B by Nexperia. It is a 14-stage ripple carry binary counter which is directly powered by the four 1.5 V AA lithium batteries. Although it is the only component of the IoT sensor node which constantly remains active, this is not an issue at all since it absorbs only 36 µA according to its datasheet [70]. This counter has been chosen since it is cheap (its price hovers around 0.10 euros) as well as easy to use and because it has a dedicated reset pin ensuring the possibility to restart the counting process via an external signal that is provided by the microcontroller. The counter drives an N-type MOSFET whose task is to switch on a 5 V step-down voltage regulator (i.e., the Pololu D24V22F5 (Pololu Corporation, Las Vegas, NV, USA)). Finally, the step-down voltage regulator powers the other parts of the sensor node: they are encompassed by Block B of Figure 1. Whenever the measurement as well as the transmission phases are completed, the microcontroller resets the counter via its dedicated pin. This entails, in turn, the interdiction of the MOSFET and the power disabling of Block B. At the same time, the counting process restarts once again involving the periodic accomplishment of the system tasks. In this way, the sensor node is provided with an energy-saving policy that will be described in Section 5.1: the counter is the only component that is constantly powered while the other ones are exclusively activated for the necessary time on any occasion they need to operate.

Concerning the oscillation frequency of the counter, the HEF4060B features two setting modes: its clock frequency could be determined both by a crystal quartz or by an RC oscillator. The latter solution was preferred since it is cheaper to implement, and bearing in mind that it is requested a measurement of the filling level of the bins per hour, the design of the RC circuit goes as follows. Since the HEF4060B is a 14-stage counter, it is capable to count up to $2^{14}-1$. Due to the fact that the N-MOSFET is controlled by the Most Significant Bit (MSB) of the counter (which corresponds to pin number 3, $Q_{13}$, of the integrated circuit) and considering that only one measurement per hour is needed, the clock frequency has to be designed in such a way that the MSB is at a high logic level after 3600 s. In other words, the HEF4060B has to count up to $2^{13}-1$ in 3600 s hence it would finish its available combinations of digits after 7200 s. Therefore, the oscillation frequency $f_{osc}$ is:

(3) $$f_{osc}=\frac{2^{14}}{7200}≃2.2756\;\mathrm{Hz}.$$

According to the HEF4060B datasheet, $f_{osc}$ has a direct relation with the components of the RC circuit ($R_{T}$ and $C_{T}$) which is:

(4) $$f_{osc}=\frac{1}{2.3R_{T}C_{T}}.$$

A 680 nF plastic film capacitor was chosen for $C_{T}$. Such a value is due to the fact that the datasheet suggests $C_{T}$ > 100 pF and to the fact that a pretty low frequency is desired. Therefore, by equaling Equations (3) and (4), $R_{T}≃280$ kΩ is obtained. In order to avoid a series connection of resistors, a commercial value was selected for such a component, hence $R_{T}$ = 270 kΩ. At this stage, by employing Equation (4), the actual clock frequency $f_{ck}$ can be evaluated: $f_{ck}≃2.3681$ Hz. As a direct consequence, the HEF4060B counts up to $2^{13}-1$ in approximately 3460 s which roughly correspond to 57 min. Such a sampling period for measurement and transmission is acceptable though a finer precision could be achieved by employing, for instance, a trimmer in place of $R_{T}$. The only two components left to design are $R_{2}$ and $C_{2}$. As stated in the HEF4060B datasheet, both this relations have to hold:

(5) $$\left\{\begin{array}{c} R_{2}C_{2}\ll R_{T}C_{T}\\ R_{T}\ll R_{2} \end{array}\right..$$

Therefore, in order to satisfy Equation (5), $R_{2}$ and $C_{2}$ were selected as follows: $R_{2}=$ 820 kΩ and $C_{2}$ = 100 pF as a ceramic capacitor.

The described system clearly has a fixed oscillation frequency which leads to a fixed sampling rate of the filling level of the trash bin. As it has been just mentioned, the usage of a trimmer in place of $R_{T}$ allows the tuning of the oscillation frequency. In so doing, the sampling rate of the filling level would be also made customizable by the users. Since the conditions expressed in Equation (5) must be always satisfied, the resistance value of the trimmer should remain within a 10 kΩ ÷ 300 kΩ range. For this purpose, a series between a 10 kΩ resistor and a suitable trimmer should be set up. It can be easily checked that the extreme oscillation frequencies are, in turn, 63.9386 Hz and 2.1313 Hz respectively implying ∼2 min and ∼64 min sampling periods.

As it was already mentioned, when the MSB raises its logic level, the N-MOSFET behaves as a short circuit. Indeed, in this application it is exploited as a mere switch controlled by the counter. As a result, the Pololu D24V22F5 supplies the other parts of the sensor node (i.e., Block B). In spite of its expensiveness (its price hovers around 8 euros), the Pololu D24V22F5 was chosen because it is reliable and efficient. Moreover, it can accept a wide range of input voltages (up to 36 V) [71]. There are also some additional features of this regulator that justify its choice though, especially in comparison with a standard linear one (i.e., a 78xx-type). Indeed, the main difference between these two categories of devices resides in the way they accomplish regulation. First of all, let us remind that power is a conservative quantity hence it has to be accounted in all its contributes throughout the regulation process. Since the input voltage is usually greater than the output one, then an energy conversion process (like the one performed by regulators) is mainly characterized by three quantities: an input power ($P_{i}$), an output power ($P_{o}$) and an excess power ($P_{e}$). It could be summarized in:

(6) $$P_{i}=P_{o}+P_{e}.$$

A 78xx-type linear regulator converts the input voltage into the output voltage by maintaining constant the current. Due to the fact that these regulators are not featured with any storage component, then $P_{e}$ turns out to be converted into heat and therefore wasted. On the other hand, the Pololu regulators are switching regulators, hence they have a control circuitry and other components among which there are also the storage ones like inductors and capacitors. The control circuitry is in charge to turn the device on and off storing energy in inductors and capacitors so to ensure a steady output voltage. Practically speaking, the regulator is switched on long enough to charge the storage components up and to provide the desired output voltage, then it is shut down letting capacitors and inductors discharge feeding their stored energy to the output. As soon as the output voltage drops too low, then the regulator is switched on once again. As a direct consequence, $P_{e}$ is stored rather than converted into heat, which implies a greater efficiency. Therefore, due to these features, a switching regulator is anyhow worthy in spite of its pretty high price.

Once Block B is switched on, the ATmega328 takes control of the sensor node being in charge of managing the measurement and the transmission stages. When they are fulfilled, the microcontroller sends the reset signal to the counter.

### 4.5. Switching and Powering System for LoRa Module

**Figure 5:**
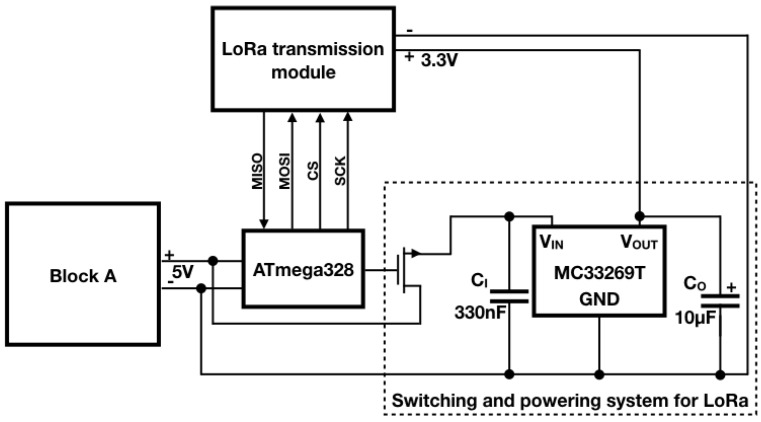
Diagram of the Switching and powering system for LoRa block.

The Switching and powering system for LoRa is in charge of properly supplying the LoRa transmission module (see Figure 5: for the sake of clarity, also additional blocks strictly related to the functioning of the one described in this subsection are included in the picture). Indeed, while Block A of Figure 4 provides the node with 5 V, that are needed for running the ATmega328, the LoRa module would be permanently damaged if fed with such a voltage due to the fact that it requires only 3.3 V. This task is fulfilled by the MC33269T voltage regulator produced by ON Semiconductor (Phoenix, AZ, USA). Moreover, this block is provided with an N-type MOSFET controlled by the ATmega328. This means that the latter is capable of switching on and off the voltage regulator, and consequentially the LoRa module, depending on when it is strictly needed (i.e., only during the transmission phase). This expedient allows further energy saving as it will be shown in the energy-saving policy Section 5.1. Likewise as it was in the previous subsection, the MOSFET is exploited as a bare switch controlled via firmware by the microcontroller by means of one of its digital outputs: once the filling level is measured, then the ATmega328 raises the logic level of the digital output connected to the gate of the MOSFET, leading to its saturation, which runs the voltage regulator allowing, in turn, the activation of the LoRa module and the transmission of the sensed data.

The MC33269T is a linear 3.3 V voltage regulator which was installed as recommended in its datasheet [72]. Indeed, it requires two capacitors that act as low pass filters: a 330 nF plastic film capacitor (i.e., $C_{I}$) and a 10 µF electrolytic one (i.e., $C_{O}$). Still according to the datasheet, it has a dropout voltage of 1 V and since its input voltage is directly provided by Block A, hence the input voltage is 5 V, no supplementary problems arise during the supplying of the LoRa module.

On account of the list of benefits offered by Pololu-like regulators (or generally by switching regulators) presented within the last subsection, one could wonder why such devices were not exploited as a regulator for the LoRa module too. For sure, a component like those would have been extremely effective from the energy-saving point of view. However, they are quite expensive (the price of the Pololu D24V22F hovers around 8 euros) and since one of the goals of the design of this IoT node is the cheapness of the node itself, then a cheaper voltage regulator (i.e., the MC33269T whose cost hovers around 0.7 euros) has been chosen even though it is not as efficient as the step-down ones: as it was explained in Section 4.4, the MC33269T has a higher consumption than the Pololu D24V22F5. Despite this, the overall energy requirements of the IoT node do not experience significant increase since (as it will be shown in Section 5.2) the MC33269T has a shorter running time than the Pololu D24V22F (thank to the energy-saving policy, see Section 5.1).

## 5. Energy-Saving Policy and Consumption Estimation

This section covers two relevant characterizing themes of the IoT sensor node effectiveness assessment: the description of the energy-saving policy and the estimation method adopted to evaluate the overall consumption and therefore the lifetime of the four 1.5 V AA lithium batteries supplying the node.

### 5.1. Energy-Saving Policy

**Figure 6:**
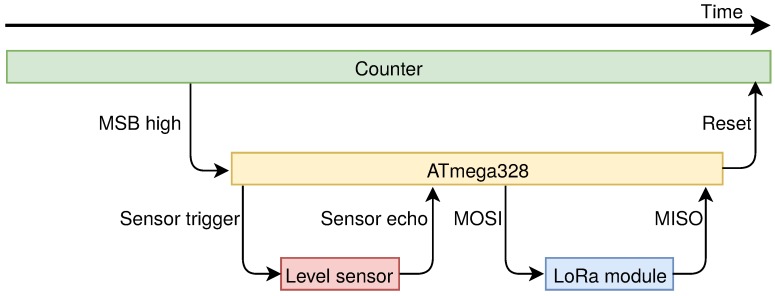
Flow chart of the energy-saving policy.

In order to facilitate the explanation of the energy-saving policy, let us refer to the flow chart in Figure 6. First of all, the chart qualitatively reports throughout a single measurement sampling period, which approximately is 57 m due to the oscillator circuit of the HEF4060B as explained before, the activation time spans of the main components of the node: the counter (i.e., the HEF4060B (Nexperia, Nijmegen, The Netherlands)) as a green bar, the ATmega328 (i.e., the microcontroller) as a yellow bar, the level sensor (i.e., the HC-SR04 (Elecfreaks, Shenzhen, China)) as a pink bar and the LoRa module (i.e., the SX1272 (Semtech, Camarillo, CA, USA)) as a blue bar. For the sake of clarity, note that the time axis is not linear: the overall time in which the ATmega328 is on, is no more than few tens of seconds. Finally, the arrows represent the events driving the activation as well as the deactivation of the single devices. Their actual meaning will be clearer as soon as the policy will be described. This policy is cyclically actuated by means of both the firmware installed on the microcontroller and some hardware component like the MOSFETs and the counter as well. Such a procedure could be summarized in the following points and, with the purpose of getting better the dynamics of this energy-efficient strategy, it is advisable to refer to Figure 1, Figure 4 and Figure 5 along with Figure 6.

1. The HEF4060B starts to count as soon as the four AA lithium batteries are installed in their slot since it is directly connected to them. Then, after 57 m, the MSB of the counter is at a high logic level implying the activation of the MOSFET within Block A. As a result, the Pololu D24V22F5 is switched on entailing the supplying of the microcontroller within Block B. The firmware installed on the chip ensures that none of the other components within Block B are activated during this phase of the policy. Indeed, the Switching and powering system for LoRa block as well as the level sensor are connected to digital output pins of the ATmega328 that provide a low logic level according to the executed firmware instruction so far.
2. At this stage the ATmega328 takes control of the node while the HEF4060B still continues to count. These two phenomena take place in parallel since they are not in conflict. Indeed, the only trouble would arise only if the MSB will lower its logic level due to the counting process: this event would cause an unwanted deactivation of the ATmega328. Fortunately, this episode will never occur since it naturally would after 57 m while the time needed for the measurement and the transmission of the data is by far shorter.
3. The level sensor is directly connected to the microcontroller which manages its activation and the acquisition of the sensed filling level too. Indeed, the supply voltage of 5 V for the sensor is provided by a digital output of the ATmega328. When such output is kept to a high logic level, due to the relative firmware instruction, then the sensor is activated. Furthermore, the firmware commands the sensor via the Sensor trigger and Sensor echo pins: the former receives the request by the ATmega328 for the measurement while the latter returns such data to the microcontroller. Once this process ends, the ATmega328 switches the sensor off by lowering the digital output devoted to the sensor powering.
4. A similar plan is implemented for the LoRa module even though it presents some slight dissimilarities. The microcontroller drives the MOSFET within the Switching and powering system for LoRa block through a digital output: raising its logical level implies the activation of the transmission module (by the way, here it is highlighted the importance of the Switching and powering system for LoRa: a feeding method specular to the one exploited for the sensor is no longer viable since the ATmega328 high logic level coincides with 5 V which will damage the LoRa module. Moreover, the microcontroller would not be able to provide the power requested for the transmission on its own). As soon as the module is turned on, then the ATmega328 firmware arranges the transmission by means of the SPI bus (MISO and MOSI signals for data and CS and SCK for controlling the communication) and when it is over, it turns off the module lowering the logic level of the pin connected to the aforementioned MOSFET.
5. At this point, the tasks of the IoT node (i.e., the measurement and transmission of the bin filling level) are accomplished. Therefore, the microcontroller, via firmware, raises the logic level of the digital pin connected to the Master Reset input of the HEF4060B (i.e., pin 12) involving the reset of the counting process which obviously means that the MSB logic level lowers resulting in the deactivation of the ATmega328. Since the counting process has just restarted, due to the fact that the HEF4060B is constantly powered, the IoT node is starting at the same time another functioning cycle.

For a matter of completeness, the actual period of the policy can be evaluated. A cycle is mainly made up of two parts: the first one which corresponds to 57 m within which the MSB is at a low logic level, and the second one which roughly corresponds to 30 s within which the MSB is at a high logic level allowing the measurement and the transmission. The extend of the second part of the period was empirically measured: there exist ad-hoc firmware instructions consenting the measurement of the time spent by the microcontroller to carry out its tasks. It was found out that, on average, 30 s are enough to guarantee the proper functioning of the microcontroller. In other words, the yellow bar in Figure 6 is 30 s length. Therefore, a single sampling measurement period $T_{m}$ has a duration of 3460 s and 30 s: $T_{m}$ = 3490 s. This figure will turn out to be useful in the following subsection.

### 5.2. Estimation of the Lifetime of the Batteries

In order to obtain an estimate of the lifetime of the batteries, at first an evaluation of the node consumption is needed. Such a consumption will be computed by accounting the nominal absorption of the main components of the node, according to the relative datasheets, as well as the time they spend running according to the policy. Passive components as well as capacitors and MOSFETs could be considered having a negligible contribution to this estimate. All these data is reported in Table 2.

Notice that the counter, the only component permanently activated, has a running time per period equal to $T_{m}$. As explained earlier on, the microcontroller has a running time per period equal to 30 s and because it is supplied by the switching regulator, also the latter must have the same activation time. Concerning the nominal consumption of the ATmega328, it is equal to 10 mA due to the fact that its clock frequency was set to 16 MHz: if it was set to 8 MHz by using an appropriate quartz crystal, then the consumption would be halved. By the way, the clock frequency might be set to the lower one since the sampling measurement frequency is by far inferior. Anyway, the clock frequency was set to 16 MHz so to maximize the microcontroller performances. The level sensor has a running time per period equal to 5 s so to perform three consecutive measurements. Then, the microcontroller computes the mean among these data achieving a better accuracy. After some empirical transmission tests, it was found out that 10 s, on average, are sufficient to ensure a correct transmission by the LoRa module driven by the microcontroller. Therefore, the linear regulator must be activated for the same amount of time since it powers the LoRa module. The consumption of the transmission module is 28 mA since it do not operate in boost mode. Indeed, after some tests, the transmission distance range was successfully covered without resorting to such expedient.

At this point, the node consumption per cycle ($c_{t}$) can be evaluated as:

(7) $$c_{t}=\frac{c_{1}t_{1}+\left[\left(c_{2}+c_{3}\right)t_{2}\right]+c_{4}t_{3}+\left[\left(c_{5}+c_{6}\right)t_{4}\right]}{T_{m}}≃0.29\;\mathrm{mA}.$$

On purpose to ease the treatise, it is better to evaluate the hourly node consumption (*c*):

(8) $$c=\frac{3600c_{t}}{T_{m}}≃0.299\;\mathrm{mA}.$$

Considering that the capacity of the four 1.5 V AA lithium batteries is 3500 mAh, then the lifetime of the batteries (*L*) can be computed as:

(9) $$L=\frac{3500}{c}≃12,040\;h,$$

which approximately corresponds to 502 days. If comparing this result with the achievable lifetime using a prototype composed by an Arduino UNO board instead of the ATmega328 microcontroller (the one shown in Figure 1 where the microcontroller block is substituted with the Arduino UNO), it is possible to appreciate the effectiveness of the proposed solution. Indeed, the absorption of current of the Arduino UNO, that was measured (by means of a digital multimeter) by powering it via a laboratory DC power supplier, is $c_{Arduino}≃38$ mA. Then, it is possible to evaluate the node consumption per cycle ($c_{t}$) in this configuration as:

(10) $$c_{t}=\frac{c_{1}t_{1}+\left[\left(c_{2}+c_{Arduino}\right)t_{2}\right]+c_{4}t_{3}+\left[\left(c_{5}+c_{6}\right)t_{4}\right]}{T_{m}}≃0.53\;\mathrm{mA},$$

that leads to the following hourly node consumption (*c*):

(11) $$c=\frac{3600c_{t}}{T_{m}}≃0.546\;\mathrm{mA}.$$

Assuming the use of the same four AA 1.5 V 3500 mAh lithium batteries, the lifetime becomes:

(12) $$L=\frac{3500}{c}≃6206\;h,$$

which approximately corresponds to 259 days, roughly half the value obtained with the proposed solution. Moreover, the average cost of an Arduino UNO board is around 20 euros, while the ATmega328 microcontroller costs around 2 euros: this means that, besides the doubled life time, the overall cost of the sensor node is one order of magnitude lower than the Arduino-based one.

This estimate strongly confirms the effectiveness of the energy-saving policy even because further improvements could be adopted. One of these could be lowering the clock frequency of the microcontroller down to 8 MHz. Another one could be the employment of more efficient hardware components. However, this would require additional studies and researches to be carried out and at the same time it could mean higher costs to be afforded for the implementation of the IoT node.

## 6. Operation Tests

The testing phase of the IoT node focused on two different tasks: proving the effectiveness of the ultrasound sensor for the measurement of the filling level and proving data transmission through LoRa channel.

### 6.1. Tests on the HC-SR04 Ultrasound Sensor

**Figure 7:**
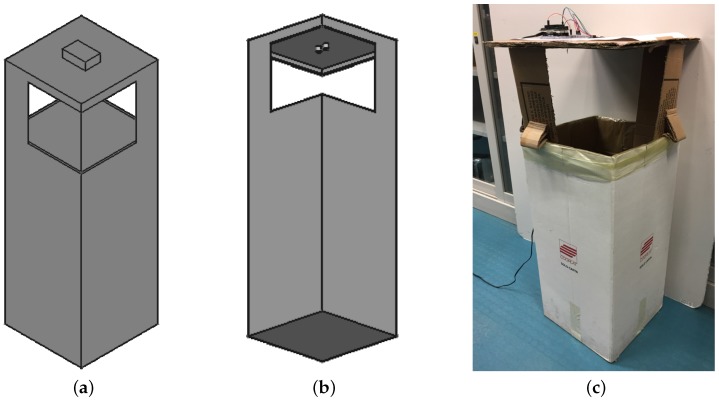
Experimental setup for the validation of the sensor. 3D model of the sensing structure: (**a**) axonometric view from above; (**b**) axonometric view from below; (**c**) final realization.

**Figure 8:**
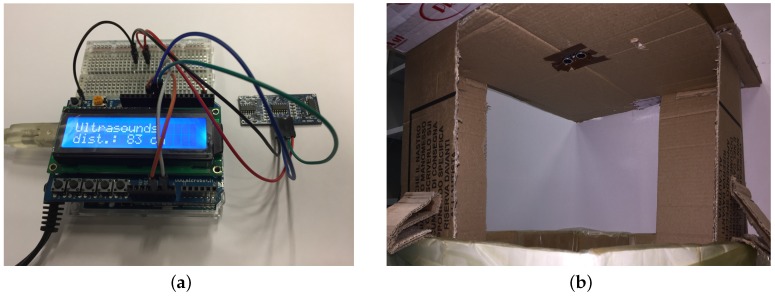
Experimental setup: (**a**) the LCD screen showing a measured distance value; (**b**) the ultrasound sensor placed in the cardboard cover.

The tests on the effectiveness of the sensor were performed by using a sub-set of the node prototype made up only by the Arduino UNO board and the HC-SR04 sensor since the presence of other components would have had no influence on the accuracy of the measurement. In addition, since an Arduino UNO board is equipped with the same microcontroller employed in the node, such a board was chosen for these tests due to its simple usage whenever rapid prototyping is needed and because the same firmware of the IoT node could be exploited. The tests were performed using a real trash bin equipped with a cardboard cover ad-hoc built with the sensor embedded in it (Figure 7). In order to ease data reading, an LCD screen was added on the Arduino UNO board displaying the distance in cm measured by the sensor (Figure 8a). The transmitter and the receiver of the sensor were directed toward the bottom of the bin, measuring then the distance of the upper surface of the garbage layer from the lower surface of the cardboard cover (Figure 8b): that distance is ∼100 cm if the bin is empty, with an approximation range of ±2 cm due to the presence of the trash bag (as in a real scenario) that could be crumpled and not perfectly adherent to the bottom of the bin.

The accuracy of the measurement with the empty bin was in the order of ∼1 cm. The trash bin was then progressively filled up with rubbish (mainly paper), and the value acquired by the sensor then compared with the distance from the sensor to the trash level measured with a measuring tape. The filling level was obtained by performing three consecutive measurements, then a mean of these data was computed by the microcontroller and finally displayed on the LCD screen. Although the value obtained through the sensor fell within a 1 cm error range by measuring the distance with the measuring tape, it did not take into account possible rubbish accumulations on the sides of the bin since the level value was measured in its central point. Anyway, due to the reduced internal surface of a trash bin compared to a dumpster, the level of the rubbish can be considered almost constant with variations lower than 5 cm. Moreover, it has to be underlined that, to asses the filling of the bin, 5 cm variations can be considered negligible.

The accuracy of the sensor was confirmed for almost the whole height of the bin. Inaccurate readings occurred only in the last 5 cm by the proximity of the upper inner surface of the bin: this value was higher than the one expected according to the sensor specifications (2 cm to 400 cm measurement range). Anyway, this result does not affect the effectiveness of the IoT node because when a 5 cm distance is measured, the trash bin can be considered full.

### 6.2. Tests on Data Transmission through LoRa Channel

The effectiveness of the LoRa technology for the deployment of a city-scale monitoring infrastructure was analyzed by testing the actual data transmission ranges in a real scenario. For this purpose, a prototype network infrastructure was set up in the historic centre of Siena, Italy, with the aim of demonstrating the feasibility of the realization of a single star network covering a whole urban area. The prototype infrastructure was constituted by a gateway node composed by a Libelium SX1272 LoRa module (Libelium, Zaragoza, Spain) connected to a laptop through a Waspmote USB Gateway and by one sensor node that will be described in a while. The three key parameters to be set for the LoRa communication modules, i.e., the Spreading Factor (SF, the number of symbols sent per bit of information), the Bandwidth (BW) and the Coding Rate (CR), were set according to the datasheet to obtain the highest sensitivity: in particular, setting SF=12, BW=125 kHz, and CR=4/5 the sensitivity value grows up to −136 dBm.

Following the network setup, the transmission ranges were tested by placing the gateway in a fixed position, and checking that the actual packet was correctly received as the sensor node was moved in different spots around the city. In particular, since the historic centre of Siena is still surrounded by its medieval city walls, the received packet was tested in a set of spots along the walls track and along the main roads of the centre, as well as in a couple of more distant sites from the centre but in line of sight with the gateway location. The gateway was placed on the top of the tower of the University Rectorate, which is exactly placed in the centre of the city and almost equidistant from the city walls. These tests proved the feasibility of the system since the maximum achieved transmission range in the city centre (i.e., within the medieval walls) was 1.1 km. Moreover, also some peripheral spots (i.e., outside the medieval walls) were tested achieving a maximum transmission rage of 2.7 km in line-of-sight, hence strengthening the practicability of the system. Concerning the spots within the city walls, the line-of-sight was not always ensured due to the morphology of the city centre. Indeed, the centre of Siena is built atop three hills: this means that the valley bottoms are not visible from the top of the Rectorate tower. Such an issue did not affect the peripheral spots though.

**Figure 9:**
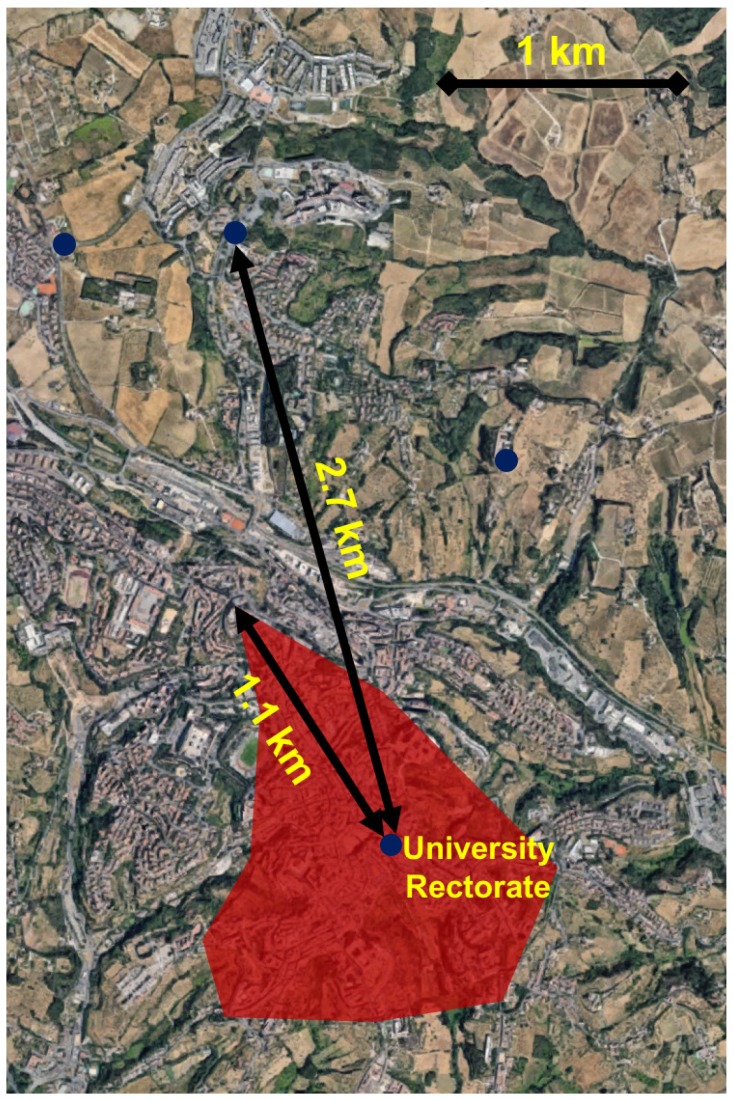
Map of the area covered by the LoRa network. Blue dots indicate the more distant line-of-sight spots.

In order to check the presence of packet losses, the data transmissions were performed exploiting a prototype composed by the HC-SR04 sensor and the Arduino UNO board provided with the Libelium SX1272 module connected through the ad-hoc Multiprotocol Radio Shield (i.e., the same prototype as the one set up in Section 6.1 equipped with LoRa connectivity). None of the packets were missed during the transmissions from all of the testing sites (i.e., at each of the city gates and every 500 m along the city walls track as well as along the main roads of the centre). Albeit packet losses were observed whenever the prototype was moved amid a testing site and the following one, this is not a matter of concern since the nodes are expected to be still on the deployed trash bins in the city centre. Thus, the LoRa technology proved to cover the system deployment area (i.e., the city centre), as it can be seen in Figure 9, without the necessity to enable the boost mode on the transmission module.

In addition, it was also empirically proved that no more than 10 s are requested to accomplish the transmission. No analysis on the received signal strength indication (RSSI) values was carried out because the main purpose of the test was only to prove the effectiveness of the data receiving and not the signal sensitivity.

## 7. Conclusions and Future Works

The aim of this paper was to present an energy efficient solution for waste management in the wider context of Smart Cities. In particular, the solution described in this paper focuses on the realization of a system in charge of measuring the filling level of trash bins and transmitting in real time the collected data to a remote data collection centre. While developed by using off-the-shelf devices, the described solution is conceived with a specific focus on the network architecture and on the optimization of power consumption as well.

Regarding the network architecture, this is based on the LPWAN LoRa technology, that proved to provide the best trade-off in terms of power consumption and performances. In particular, LoRa radio modules turn out to be energy efficient, with reduced consumption in transmission if compared to similar technologies: LoRa modules current absorption in transmission is around 20 mA while, for example, off-the-shelf ZigBee radio modules, such as XBee Series 2 absorb, according to the datasheet, around 40 mA. They are characterized by wide area transmission ranges that allow to set up urban-area networks by employing a single or few access points. While these networks are based on star topologies, they allow to apply strict duty-cycling policies to the sensor nodes with an additional, notable reduction on power consumption. The data transmission ranges and the effective feasibility of a single gateway, along with a city-scale network were confirmed by the tests carried out: these tests showed that a 1.1 km transmission range is achievable in urban areas even without line-of-sight, while if ensuring line-of-sight, this value could grow up ∼3 km.

Regarding the power optimization, by using low power components as well as by combining duty-cycling policies, the developed IoT node is expected to operate for an ideal span of time up to 502 days according to power consumption analysis and preliminary laboratory tests. This value is expected to be confirmed with the actual implementation of the system: direct long run field tests were not carried out though due to the high variability of the environmental conditions of the deployment scenario. Anyway, the system is conceived with a lifetime that, even its half, widely ensures the operation of the whole monitoring infrastructure. Moreover, this value has been compared with the one achievable by developing the same sensor node using an Arduino UNO board: calculations have shown that the achieved lifetime (502 days) is roughly twice than the one obtainable with the Arduino-based node (259 days).

Together with the extended lifetime, the proposed sensor node is also characterized by reduced costs. The main components (microcontroller, counter, sensor, voltage regulators), excluding the LoRa module account for around 10 euros. The LoRa module used for this prototype (i.e., the Libelium SX1272) has currently high costs (around 40 euros), but cheaper alternatives can be found and are emerging day-by-day. Future work will be carried out in this direction, in order to identify cheaper alternatives providing a similar performance level.

Additional future work is expected to be carried out also to improve the efficiency of the node. In particular, possible directions to be investigated include the use of two different battery sets to power separately Block A and Block B (see Figure 1) and the adoption of additional software-based energy efficient policies. Some examples could be the use of adaptive protocols modifying the sampling rate according to the variation rate of the filling level of the trash bins as well as transmission policies activating the LoRa module only when significant level variations are detected.

In conclusion, the described IoT node was developed and tested in laboratory for the validation of the measured data and in field for the data transmission, proving its effectiveness. The overall architecture is expected to be deployed in a real scenario in the next future: anyway, this is strictly dependent on the policies carried out by the local waste management companies.

## Figures and Tables

**Table 1:** SX1272 module characteristics.

Parameter	Value
Frequency Band (Europe)	863–870 MHz
Frequency Band (US)	902–928 MHz
Transmission Power	14 dBm
Sensitivity	−134 dBm
Channels (Europe)	8
Channels (US)	13
Distance	22 km (13.4 mi)

**Table 2:** Nominal consumption of the devices composing the node and their relative running time within $T_{m}$.

Device Type	Device Name	Nominal Consumption	Running Time
Counter	HEF4060B	$c_{1}$ = 0.036 mA	$t_{1}$ = 3490 s
Switching regulator	Pololu D24V22F5	$c_{2}$ = 1 mA	$t_{2}$ = 30 s
Microcontroller	ATmega328	$c_{3}$ = 10 mA	$t_{2}$ = 30 s
Level sensor	HC-SR04	$c_{4}$ = 15 mA	$t_{3}$ = 5 s
Linear regulator	MC33269T	$c_{5}$ = 20 mA	$t_{4}$ = 10 s
LoRa module	SX1272	$c_{6}$ = 28 mA	$t_{4}$ = 10 s
